# Exploring the Role of Green Microbes in Sustainable Bioproduction of Biodegradable Polymers

**DOI:** 10.3390/polym15234617

**Published:** 2023-12-04

**Authors:** Adenike Akinsemolu, Helen Onyeaka

**Affiliations:** 1 Institute of Advanced Studies, University of Birmingham, Birmingham B15 2TT, UK; 2 Department of Integrated Science, Adeyemi Federal University of Education, Ondo 351101, Nigeria; 3 School of Chemical Engineering, University of Birmingham, Birmingham B15 2TT, UK

**Keywords:** green microbes, biodegradable polymers, sustainable bioproduction, polyhydroxyalkanoates, metabolic pathways

## Abstract

Research efforts have shifted to creating biodegradable polymers to offset the harmful environmental impacts associated with the accumulation of non-degradable synthetic polymers in the environment. This review presents a comprehensive examination of the role of green microbes in fostering sustainable bioproduction of these environment-friendly polymers. Green microbes, primarily algae and cyanobacteria, have emerged as promising bio-factories due to their ability to capture carbon dioxide and utilize solar energy efficiently. It further discusses the metabolic pathways harnessed for the synthesis of biopolymers such as polyhydroxyalkanoates (PHAs) and the potential for genetic engineering to augment their production yields. Additionally, the techno-economic feasibility of using green microbes, challenges associated with the up-scaling of biopolymer production, and potential solutions are elaborated upon. With the twin goals of environmental protection and economic viability, green microbes pave the way for a sustainable polymer industry.

## 1. Introduction

Biopolymers are a type of polymers that are produced from natural sources and are either synthesized chemically from biological material or by living organisms using enzymes that link natural sources such as sugars, amino acids, hydroxyl fatty acids, and other building blocks to produce polymeric materials [1]. The versatility of biodegradable polymers has positioned them as a great and viable alternative to conventional plastics, which are typically non-biodegradable and toxic, leading to their application in several industries, including the pharmaceutical, cosmetic, food, agriculture, mining, printing and textile, oil recovery, and waste management industries [2]. As environmental protection and economic viability become primary concerns in production processes, the use of conventional chemical stabilizers in the production of polymers has become less viable. Therefore, it has become increasingly important to explore different ways of enhancing the sustainability of the use of algae, bacteria, and fungi to produce biodegradable polymers [3]. To this end, this review synthesizes existing knowledge in biodegradable polymers to explore different microbes and their roles in the production of biodegradable polymers, with specific emphasis on the potential for the genetic engineering of the organisms and the processes they use to form biopolymers to increase their production yield, the potential challenges in this process of scaling up the production of biopolymers, and viable solutions to these challenges to guarantee a sustainable biopolymer industry. 

## 2. Materials and Methods

A systematic review was employed to provide a comprehensive perspective of the role of green microbes in the sustainable production of biodegradable polymers. Data for this study were extracted from PubMed, ScienceDirect, and Google Scholar, which were chosen for their comprehensive collections of sources on the topic of study. To minimize bias and guarantee consistency, all data were collected on a single day. The keywords used in the search were “biopolymer production”, “microbial biopolymer production”, “economic viability of biopolymer production”, and “sustainability in biopolymer production”. All documents that met the search criteria, were published between 2015 and 2023, and were available in English were selected, their abstracts analyzed for relevance to the topic of study, and duplicates eliminated. The remaining sources were read in depth, information extracted from them, and synthesized for use to inform the assertions made in the current study. 

## 3. Results

### 3.1. Green Microbes and the Production of Biodegradable Polymers

#### 3.1.1. Bacterial Biopolymers

**Scheme 1:**
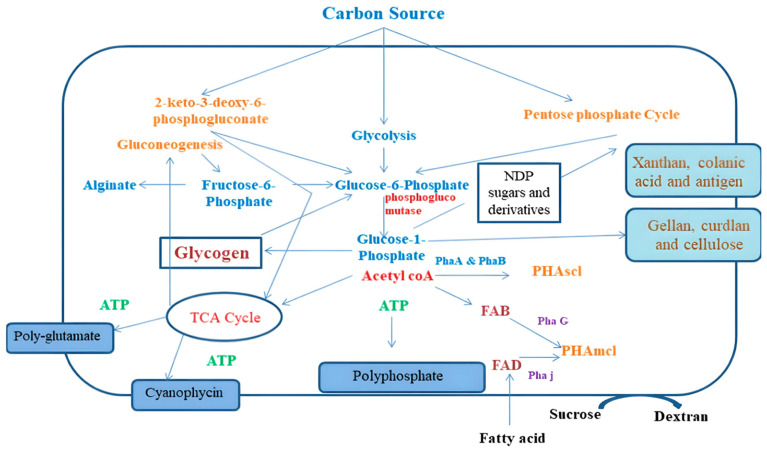
The Mechanism of Bacterial Biopolymer Synthesis Pathways [1].

Bacteria are highly adaptable microorganisms that can survive in a diverse range of ecosystems [4]. Therefore, they can synthesize multiple classes of biopolymers, including polysaccharides, polyamides, and polyesters [4]. The role of bacteria in the production of bacterial polymers and the types of biopolymers that are synthesized by bacteria are well documented in Table 1. Polysaccharides are high-molar-mass polymers that are produced from sugars and sugar acids. The typical metabolic pathway to produce biopolymers involves three enzymatic actions (Scheme 1). In the first step, ADP-Glucose pyrophosphorylase (AGPase) catalyzes adenosine diphosphate-glucose (ADP-Glc). In the second step, linear 1-1,4-linked glucose chains are generated with glycogen synthase as the catalyst. The chains give glycogen its helical structure, making it a suitable energy reserve. Finally, a-1,6-linked glucan branches are produced in the polymer, with a branching enzyme as the catalyst [5]. The resulting biopolymers are easy to use and more sustainable compared to their more conventional chemical alternatives.

The biopolymers are produced and stored inside cells to serve as carbon and energy reserves [6]. Due to their high molar mass, biopolymers, such as polysaccharides, constitute biofilm matrices with diverse material properties that make them suitable for use in different materials. Depending on their material properties, such as the type of glycosidic linkages, length of the polymers, solubility, ionic strength, extendibility, and molar mass, bacterial polysaccharides have a wide range of applications in the manufacture of natural viscosifiers and thickeners, gel, microcapsules, foams and fibers, nanoparticles and nanotubes, and sponges, with applications in drug delivery, cosmetics, and biomedical and food packaging [4]. The bacterial species that are commonly used in the production of polysaccharides include *Acetobacter polysaccharogenes*, *Acetobacter xylinum*, *Pseudomonas aeruginosa*, *Bacillus thuringiensis*, *Escherichia coli*, *Pasteurella multocida*, *Lactobacillus acidophilus*, and *Geobacillus thermonitrificans* [6]. 

**Scheme 2:**
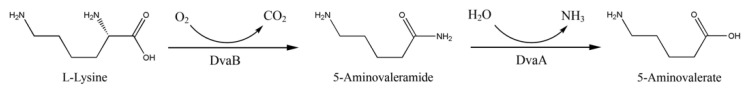
The AMV Pathway [8].

While the most abundantly produced and used polysaccharides are glycogen, cellulose, and starch, which are connected by glycosidic linkages, polyamides are made up of amino acids that are linked through peptide bonds [4]. Most biopolyamides are either co-polymers of diacids and diamines or homopolymers of terminal amino acids [7]. One metabolic pathway for the synthesis of biopolyamides produces amino acids such as 5-AVA as an intermediate in the process of degrading L-lysine through the AMV pathway using *Pseudomonas putida* or *Escherichia coli* as the source of carbon and nitrogen (Scheme 2) [8].

The primary source of the bio-based monomers used in the production of biopolyamides is castor oil, which contains an abundant mixture of saturated and unsaturated fatty acids. Pressed castor oil is converted to octanedicarboxylic acid, decanediamine, and aminoundecanoic through pyrolysis. In the final step, the monomers are polymerized into biopolymyadies [7]. In addition to *Pseudomonas putida*, *Xanthomonas campestris*, *Corynebacterium glutamicum*, and *Escherichia coli* are used in the production of biopolyamides. 

**Scheme 3:**
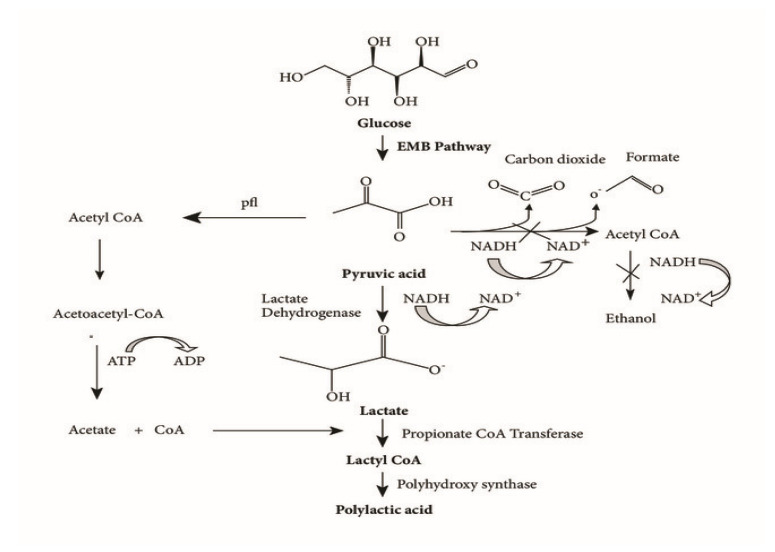
The Process of the Bioproduction of Polylactic Acid [10].

Bacteria are widely used in the production of biopolyesters as well, among them Polyhydroxylalkanoates (PHAs). PHAs are preferred to their synthetic chemical alternatives due to their biodegradability [9]. There are different metabolic pathways for the synthesis of polyester based on the bacteria strain used in the production of the materials. For instance, the process of the manufacture of polyester using lactide begins with the condensation of lactic acid to form a pre-polymer with low metabolic weight. In the second step, the pre-polymer undergoes polymerization to produce lactide, which then undergoes ring-open polymerization to form Polylactic Acid, a type of polyester (Scheme 3).

*Lactobacillus bulgaricus*, *lactobacillus delbrueckii*, and *lactobacillus leichmannii* are used in the fermentation process in the production of the monomers that are polymerized in the second step to form the monomers that undergo ROP to produce polyester [11]. The process yields polylactic acid (PLA), a type of biopolyester. Other bacteria used in the production of PHAs include *Actinobacteria*, *Bacteroidetes*, *Cyanobacteria*, *Proteobacteria*, *Clostridia*, and *Bacilli* [11]. Evidently, bacteria provide a wide variety of polymers with extensive technical, industrial, and medical applications. Specific applications include drug delivery, wound dressing, as an additive in reconstructed foods such as cake mixtures and frozen custards, and in the manufacture of regenerative medicines [12]. 

#### 3.1.2. Fungal Biopolymers

The literature on fundal biopolymers recognizes the two primary properties of fungi, on which different industries have capitalized on to produce biopolymers using the microorganisms; they are fast growing and can convert nutrients from low-cost and low-value waste into biopolymers [13]. Consequently, fungal waste from wineries, which includes saccharomyces and non-saccharomyces yeasts, waste from enzyme production, which includes *Aspergillus* and *Trichoderma, penicillium* waste from the manufacture of antibiotics, and waste from the mushroom industry are up-scaled for use in the production of fungal exopolysaccharides such as Aureobasidium, Sclerotium, and Botryosphaeria [14].

**Scheme 4:**
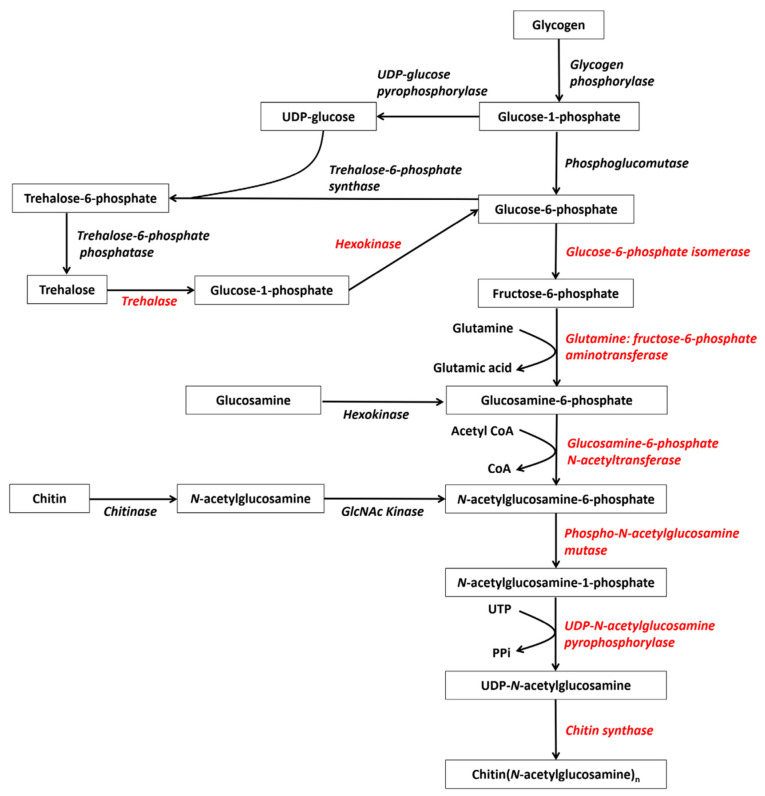
The Metabolic Pathway of Chitin [17].

After cellulose, the second-most abundant biopolymer is chitin, a carbohydrate-based component of the cells of fungi [15]. The metabolic pathway for the synthesis of chitin is a seven-step process that begins with glycogenolysis, which breaks down glucose into glucose-1-phosphate and glucose (Scheme 4). After the initial phosphorolysis, a second catalysis occurs in the presence of phosphomutase. The compounds in the subsequent steps are broken down progressively until chitin is formed in the last step [16]. 

Chitin and its derivative, chitosan, are easily recovered from fungal classes such as *Absidia coerulea*, *Absidia glauca*, *Absidia blakesleeana*, *Mucor rouxii*, *Aspergillus niger*, *Phycomyces blakesleeanus*, *Trichoderma reesei*, *Colletotrichum lindemuthianum*, *Gongronella butleri*, *Pleurotus sajocaju*, *Rhizopus oryzae*, and *Lentinus edodes* (see Table 2). The applications of the chitin and chitosan recovered from these fungi include the production of paper and textiles, biofertilizers, wound-dressing and wound-healing management products, artificial kidney membranes, tendons, cartilage, and skin, drug-delivery systems for the delivery of vaccines, and in burn treatment [16].

#### 3.1.3. Algal Biopolymers

**Scheme 5:**
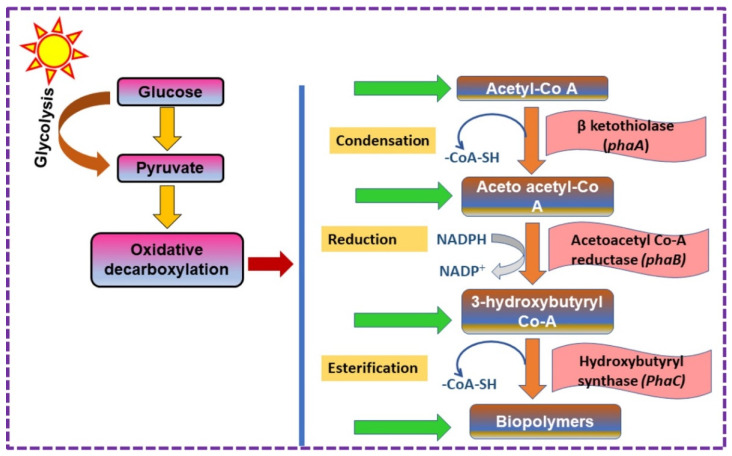
The PHB Biosynthetic Pathway [19].

Algae are photoautotrophic organisms with high biomass and a high rate of growth. The microorganisms have limited nutritional requirements, the ability to thrive in environments that are not arable and typically do not support the growth of plants, including wastewaters, and the ability to yield biomass regardless of the season, making them perfect for the production of organic compounds [18]. These qualities of algae, combined with the microorganism’s ability to assimilate carbon dioxide into different organic compounds, make them perfect materials for the manufacture of biopolymers. The most common algal biopolymers are Poly Lactic Acid (PLA) and Polyhydroxyalkanoates (PHAs) (see Table 3). The metabolic pathway for the synthesis of biopolymers from algae begins with the transformation of acetyl Co-A to acetoacetyl Co-A using 3-ketothiolase enzyme through condensation (Scheme 5). Once condensation is complete, acetoacetyl Co-A reductase reduces acetoacetyl Co-A into hydroxybutyryl. The biopolymer is formed in the third step, in which polymerization occurs in the presence of polymerase [19]. 

This process is commonly used in the production of Polyhydroxybutyrate (PHB), a polymer that is similar to petroleum-based polymers in its quality and properties and is commonly marketed as PHA. In addition to PLA, PHA, and PHB, algae are also used in the production of polyurethane (PU). PU is made from algal oils, whose composition of fatty acids are ideal for the production of similar to polyols. Polyurethane is produced through the epoxidation of algae such as Chlorella with ethylene glycol and lactic acid. The resulting polymers have good physio-mechanical properties and exhibit antibacterial and anticorrosive qualities [20]. Ultimately, algae and cyanobacteria have high nitrogen concentrations, which improves their biofilm formation. Other algal strains, besides Chlorella, that are commonly used in the production of biopolymers include Chlamydomonas, which is the best strain for the production of bioplastic, Scenedesmus, and Spirulina [20].

### 3.2. The Potential for Genetic Engineering to Augment Biopolymer Production Yields

Biopolymers have a wide range of industrial applications that are studied in depth and the potential for genetic engineering in uncovering new applications and improving the properties of biopolymers to make them better suited for these processes explored in various studies. In the medical and pharmaceutical industries, they are used in the production of drugs that protect against resistant microbes, as coatings and carriers for drugs, in the production of wound dressings, and in tissue engineering to produce artificial kidney membranes, skin, and other types of tissue. In the food industry, biopolymers are used in the manufacture of packaging, films, food additives, thickeners, stabilizers, binders, coagulants, and fillers. Biopolymers are also effective in waste management and bioremediation through their role as dye removers and flocculation agents [21]. Other industries in which biopolymers are used, and have the potential for more applications, include the automotive industry in the manufacture of vehicle interior and exterior parts, the agriculture industry in the manufacture of fertilizers, mulching films, and compostable bags, and the textile industry in the manufacture of fibers and dyes. In all these industries, biopolymers are better alternatives to synthetic polymers since they are biodegradable, do not harm the environment, and improve soil quality upon disposal [22]. Therefore, the potential for the improved production of biopolymers has attracted wide attention, leading to extensive research into ways of improving the microbial production of the polymers. 

Genetic engineering has the potential to improve biopolymer production yields. Several engineering strategies have proven effective in enhancing the production of different biopolymers. First, metabolic pathway engineering can improve the production of PHA by enhancing the synthase genes. This type of genetic engineering has been proven effective in increasing the rate and efficiency of the conversion of substrates to biopolymers [23]. Metabolic pathway engineering has been found to be effective in increasing PHA synthesis activity *in Cupriavidus necator*, *Rhodobacter sphaeroides*, *Pseudomonas putida* and other Pseudomonas strains, and *Aeromonas hydrophila*. A second form of genetic engineering, whose potential to increase the production of biopolymers using microorganisms has been proven, is co-factor and regulator engineering. This process enhances the production of different biopolymers by enhancing energy metabolism under different environmental conditions [23]. The concentrations of co-factors are adjusted under different economic conditions until the optimum conditions for the polymerization of polymers are met. Finally, promoter engineering can improve the production of bio-based polymers by rational tuning the enzymes involved in the metabolic pathways through which the polymers are produced [24]. The genetic background of the strains of microorganisms used in the production of biopolymers determines the strength of the promoters. Varying the strength of the promoters enhances the biosynthesis processes, which can increase the production of biopolymers [23]. Applying the three types of genetic engineering can increase the production of biopolymers, harnessing their benefits on sustainability, environmental conservation, and green production across the pharmaceutical, agriculture, food, medicine, textile, and other industries. 

### 3.3. Techno-Economic Feasibility of Using Microbes for the Production of Biopolymers

The benefits of biopolymers are significant and their potential to rid the planet of non-biodegradable plastics and other polymers that pollute land and water, transform waste into biopolymers, and reduce greenhouse emissions has been recognized and studied extensively. Notably, this discussion extends to evaluating the feasibility of biopolymers through near-universal concession that their continued use and the mainstreaming of genetic engineering to increase their usage is dependent on their techno-economic viability. To this end, several dynamics of the viability of the production of biopolymers are explored. First, the cost of producing biopolymers is relatively higher compared to the cost of producing petroleum-based polymers and polymers derived from biomass. As of 2022, the average price of polymers produced using feedstock and biomass was between USD 1.25 and USD 2.53. Comparatively, the cost of producing biopolymers such as PHAs was 16 times more than the cost of conventional polymers produced from petroleum. These costs were determined using energy consumption, biopolymer yield, and downstream efficiencies [25]. Although the cost of producing biopolymers is higher than the cost of all other alternatives in the market, it could be offset by other indirect benefits of the preference of the polymers over chemical-based polymers produced from petroleum and their eco-friendly alternatives produced from feedstocks and other types of biomass. These benefits include the biodegradability of biopolymers and their potential production from waste. 

The second dynamic of the techno-economic viability of the production of biopolymers is their reliance on microorganisms. Some microorganisms have low productivity and require expensive media to reproduce for use in the production of biopolymers, with the culture medium alone making up an estimated 30% of the total cost of biopolymer production [26]. For instance, some of the bacteria used in the production of PLA are produced through microbial fermentation. The process of fermentation is highly expensive, leading to the high costs associated with the microbial production of biopolymers [27]. Similarly, the bacteria used in the production and storage of PHAs and other biopolymers have relatively lower yields, and innovations to increase their yields are relatively costly, further questioning the feasibility of the use of some microbes in the production of biopolymers. However, some microorganisms are easier to grow and their growth has some benefits for the environment as well. For instance, algae, which are used widely in the production of PLAs and PHAs, are sustainable, renewable, and easy to grow, making them good sources and stores of carbon and carbohydrates. Additionally, they use inorganic compounds and transform them into proteins, lipids, carbohydrates, and other organic compounds that have a wide range of industrial applications, including the polysaccharides used in biopolymer production [28]. The ability of algae to convert inorganic compounds into organic compounds has led to their use in the reclamation of wastewater [29]. The use of algae in the bioremediation of wastewater makes their use in the production of biopolymers multi-dimensional; they are grown in wastewater for use in the production of PLAs and PHAs. Therefore, while some microorganisms used in the production of biopolymers have low yields and require expensive media to reproduce, others such as algae are more economical to grow and have significant benefits for the environment.

Third, the cost of producing biopolymers is high but the cost of their disposal is relatively low compared to the cost of plastic polymers. Biopolymers are biodegradable. They are produced using microbial activity in the presence of carbon-based materials. However, the qualities that make them biodegradable are often traded off for better functionality in attempts to match the physical and mechanical properties of petroleum-based polymers, reducing their ability to decompose fully after their use [30]. These trade-offs with functionality for biodegradability eliminate one of the justifications of the high cost of producing biopolymers, further denting the perceived feasibility and viability of the use of the polymers in place of their petroleum-based alternatives, which are cheaper to produce, have mechanical properties that biopolymers do not possess naturally, and whose raw materials are easier to source. Further, technological advancements in the development of biopolymers are made alongside similar advancements in the degradation of their alternatives, which could eliminate one of the main justifications for biopolymers over their synthetic alternatives. Fortunately, genetic engineering holds the solutions to the impediments to the feasibility of the use of microorganisms in the production of biopolymers.

### 3.4. Challenges Associated with the Up-Scaling of Biopolymer Production

The impediments to the viability of the use of microorganisms in the production of biopolymers pose several challenges in biopolymer production, which have been explored comprehensively by studies and the literature on the topic. The universal primary challenge in increasing the production of biopolymers is cost. The use of bacteria, algae, and fungi to produce PHAs, PLAs, and other biopolymers is prohibitive. PHAs are the most common substitutes for the conventional biopolymers produced from synthetic petrochemicals owing to their similarities in properties. However, the cost of producing them is significantly higher than the cost of producing synthetic polymers. In fact, cost is listed as the main barrier to the projected expansion of the market for bioplastics in Europe through 2027 [31]. The main reason for the high cost of production of biopolymers is the cost of the substrates, particularly the source of carbon used as a source of energy [32]. This cost drives up the overall production costs for most biopolymers by up to 80%. Consequently, despite efforts to increase the adoption of biopolymers, only 1% of the world’s polymers are produced using microorganisms with the rest of the polymers being based on petroleum-based polymers [21]. 

The second challenge faced in efforts to upscale the use of microorganisms for the production of biopolymers is the lower quality of the polymers compared to the conventional synthetic polymers in the market. Different microbes yield different qualities and functionalities, creating a challenge in the selection of specific microbes for the production of different polymers. For instance, PLA and PHA, which are commonly used in the food industry to manufacture packaging materials, have limited thermal and barrier properties compared to their alternatives, which are based on petroleum [1]. These drawbacks are not limited to PHAs and PLAs. Biopolymers are typically less functional owing to their poor mechanical properties, as evident in their low tensile strength and low barrier properties, which are evident in the high water-vapor permeability of packaging material produced using microorganisms [33]. While it is possible to reinforce these properties and bring biopolymers to par with their synthetic alternatives, such efforts compromise their ability to decompose completely after use, eliminating one of the primary justifications for the use of biopolymers instead of polymers produced from fossil fuels. Coupled with their high cost of production, which raises the prices of biopolymers several times above the prices of conventional polymers, these differences in the physical properties and functionality of biopolymers hinder their mainstreaming and are responsible for the fact that only 1% of the world’s polymers are produced using microorganisms, with the rest of the polymers being based on petroleum-based polymers [31].

Third, although biopolymers are positioned as the environmentally friendly alternative to synthetic polymers, they pose significant health and environmental challenges that hinder their mass adoption by eroding their perceived role as the better alternative to petroleum-based polymers. Biopolymer production has been linked to greenhouse gas emissions and negative land use changes [34]. For instance, the production of polyhydroxybutyrate (PHB) is an energy-intensive process that consumes energy of between 55 and 76 MJ for every kilogram of PHB produced and emits up to 5 kg of carbon for every kilogram of PHB produced [35]. In addition to their production, which consumes high levels of energy and emits greenhouse gases into the atmosphere, the disposal of bioplastics contributes to the global warming menage as well. Biopolymers typically require specific conditions to decompose fully. Their disposal in any other conditions not only piles on landfills but also produces methane, further contributing to global warming [36]. 

### 3.5. Potential Solutions

The three challenges of the high cost of producing biopolymers, the limited mechanical and functional properties of biopolymers, and the negative impact of their production and disposal on the environment pose significant threats to the uptake and upscaling of the production and use of biopolymers. Various studies have focused on the development of potential solutions to optimize the process of producing biopolymers to lower the cost of production, streamline the process of the growth and production of the microbial strains needed for the production of biopolymers, and reduce the negative impact of the biopolymers on the environment. One key solution to the high cost of production of biopolymers is the use of waste carbon to reduce the restrictive cost of carbon sources [32]. This can be achieved through the use of substrates derived from waste as a source of carbon and energy and reliance on microorganisms that are capable of consuming carbon and energy efficiently and using inexpensive substrates, such as waste, to produce a wide range of biopolymers. Viability studies have identified some of these strains and the inexpensive sources of energy they can use to produce the polymers. The studies confirm the viability of the use of agro-industrial bio-waste in the production of PHAs using bacteria such as *Escherichia coli*, *Pseudomonas*, *Bacillus*, and *Cupriavidus* has been proven to reduce the cost of carbon used in the process by 45%, which has been found to reduce the cost of producing biopolymers substantially [37]. Similarly, the use of photoautotrophic organisms such as algae and cyanobacteria reduces the cost of substrates significantly, since the organisms are easy and cheaper to grow and produce compared to heterotrophic bacteria. Moreover, algae can grow on wastewater, which would reduce the cost of carbon, avoid the use of arable land that is suitable for agriculture for the production of sources of carbon for the production of biopolymers, and aid in the bioremediation of wastewaters through the removal of toxins. In addition to using waste as a source of energy and carbon for microorganisms and growing algae on wastewater to reduce the pressure on land, the cost of producing biopolymers can be lowered through the manipulation of carbon substrates into forms that can be used by different microorganisms using genetic engineering [38]. Genetic engineering has proven to be effective in the expansion of the substrate spectrum by enhancing substrates to enable the production of biopolymers and enhancing the properties of the biopolymers to be identical or close to the properties of synthetic polymers. Therefore, genetic engineering has the potential to solve the problems of cost and quality simultaneously. 

Finally, the problem of the negative impact of the production and disposal of biopolymers on the environment can be solved through the proper disposal of the polymers after use. One of the most viable waste management strategies for biopolymers is recycling. Recycling biopolymers at the end of their lifecycle simultaneously solves the problems of production costs and the high cost of substrates by enabling the reuse of valuable material such as lactic acid, which can then be transformed into feedstocks for monomers and turned into new biopolymers [39]. However, the implementation of this solution will require significant consumer education, investment in sorting technology to separate biopolymer waste from regular plastic waste, and continued investment in innovation on the genetic engineering of substrates from waste.

## 4. Discussion

Evidently, biopolymers are a double-ended invention that protects the environment and have the potential to rid the planet of petroleum-based polymers but have a high cost of production and contribute to environmental degradation albeit at a much smaller scale compared to their synthetic alternatives. To harness the benefits of biopolymers fully while reducing their drawbacks, we present the following recommendations for the upscaling of their production while guaranteeing their economic viability and protecting the environment.

First, genetic engineering has the potential to optimize the qualities of biopolymers, reduce the cost of production, and establish a cycle of reuse whereby used biopolymers that are ready for disposal provide some of the substrates used in the production of new biopolymers. However, the optimization of the qualities, usability, and biodegradability of biopolymers, or their circularity, is dependent on the comparative unviability of their alternatives. As such, investments in the genetic engineering of biopolymers are only viable if their total costs of production, disposal, and reuse are lower than the total costs of the use of synthetic polymers. With advancements in technologies that use microorganisms to degrade synthetic polymers rivaling advancements in biopolymer production technologies, research, innovation, and investment in genetic engineering should focus on the enhancement of substrates to create a wide range of substrates that provide and store energy and carbon, the enhancement of the microorganisms used in the production of biopolymers to improve their ability to survive in diverse environments and multiply rapidly, and the enhancement of the properties of monomers and polymers to ensure that they match or surpass the quality of synthetic polymers. 

Second, recycling has the potential to upscale the production of biopolymers while reducing the cost of production and reducing the emission of greenhouse gases when biopolymers end up in landfills. Therefore, a good recycling strategy is key to guaranteeing the economic viability of the production and use of biopolymers while protecting the environment from the harmful synthetic polymers and the equally detrimental biopolymers if they are not disposed of properly. In addition to enhancing the circularity of biopolymer production and use, the reuse of biopolymers has the potential to drive a significant reduction in the global use and disposal of plastics, which is one of the most pressing environmental pollution problems across the world. However, the implementation of this solution requires significant investment in consumer education, the genetic modification of the microorganisms used in the production of biopolymers, and constant research and development to establish cheaper and more viable ways of producing and recycling biopolymers. 

Finally, research should focus on extending the uses of biopolymers beyond the known uses of synthetic polymers to further justify the high cost of their production. This has been achieved in the invention of new uses of biopolymers, such as the recent recognition of their potential for use in the fortification of food packaging made from biopolymers with antimicrobial technology, which extends the packaging’s purpose beyond merely packaging food to extending its shelf life and preventing its spoilage. Such upscaling will increase the value derived from biopolymers, further confirming the techno-economic feasibility of their production.

## 5. Conclusions

Biopolymers are positioned as the more environmentally friendly and overall better alternative to synthetic petroleum-based polymers. While they are biodegradable and can make use of waste, biopolymers are relatively costly to produce compared to petroleum-based polymers. Further, their ability to decompose aerobically and fully depends on the conditions under which they are disposed of. Fortunately, genetic engineering, optimal disposal, and recycling strategies, and the potential for the expansion of the applications of biopolymers, justify the current high cost of producing biopolymers while promising to make the process more economically viable and sustainable.

## Figures and Tables

**Table 1:** Types of Bacterial Biopolymers.

Type of Microorganisms	Species/Strain	Biopolymers
Bacteria	*Acetobacter polysaccharogenes*, *Acetobacter xylinum*, *Pseudomonas aeruginosa*, *Bacillus thuringiensis*, *Escherichia coli*, *Pasteurella multocida*, *Lactobacillus acidophilus*, and *Geobacillus thermonitrificans*	Polysaccharides
	*Pseudomonas putida, Xanthomonas campestris*, *Corynebacterium glutamicum*, and *Escherichia coli*	Polyamides
	*Lactobacillus bulgaricus*, *lactobacillus delbrueckii*, and *lactobacillus leichmanni*	Biopolyesters
	*Actinobacteria*, *Bacteroidetes*, *Cyanobacteria*, *Proteobacteria*, *Clostridia*, and *Bacilli*	Polyhydroxylalkanoates

**Table 2:** Types of Fungal Biopolymers.

Type of Microorganisms	Species/Strain	Biopolymers
Fungi	*Aspergillus Trichoderma*, *and penicillium*	Exopolysaccharides
	*Absidia coerulea*, *Absidia glauca*, *Absidia blakesleeana*, *Mucor rouxii*, *Aspergillus niger*, *Phycomyces blakesleeanus*, *Trichoderma reesei*, *Colletotrichum lindemuthianum*, *Gongronella butleri*, *Pleurotus sajocaju*, *Rhizopus oryzae*, and *Lentinus edodes*	Aminopolysaccharides

**Table 3:** Types of Algal Biopolymers.

Type of Organism	Biopolymers
Algae	Polylactic Acid
	Polyhydroxyalkanoates
	Polyhydroxybutyrate

## Data Availability

Data is contained within the article.
